# A case report of adult lead toxicity following use of Ayurvedic herbal medication

**DOI:** 10.1186/1745-6673-8-26

**Published:** 2013-10-02

**Authors:** Laura Breeher, Fred Gerr, Laurence Fuortes

**Affiliations:** 1 Department of Occupational Medicine, Mayo Clinic Health System, 1025 Marsh St, Mankato, MN 56001, USA; 2 Department of Occupational and Environmental Health, College of Public Health, The University of Iowa, 105 River Street, Iowa City, IA 52242, USA; 3 Occupational and Environmental Health and Internal Medicine, College of Public Health, The University of Iowa, 2207 Westlawn Bldg, Iowa City, IA 52242, USA

**Keywords:** Lead poisoning, Clinical toxicity, Ayurvedic medicine, Metals, Chelation

## Abstract

**Introduction:**

Ayurvedic medications consist of herbs that may be intentionally combined with metals, such as lead, mercury, iron, and zinc. Ayurvedic practitioners and their patients believe that the toxic properties of the metals are reduced or eliminated during preparation and processing.

**Case report:**

A 69 year old Caucasian male retired professional with a prior history of stroke presented for evaluation of new onset depression, fatigue, generalized weakness, constipation, anorexia, and weight loss. History revealed that his symptoms were temporally related to initiation of an Ayurvedic herbal medication. The patient had been previously admitted to another hospital for these symptoms and was found to have a severe anemia for which no etiology was found. Laboratory tests revealed an elevated blood lead level and a diagnosis of symptomatic lead toxicity was made. The patient was treated with intramuscular, intravenous, and oral chelation therapy to promote lead excretion. Because of complaints of continued poor mental function, neuropsychological tests were administered before and after one of the chelation treatments and showed improvement in measures of attention and other cognitive domains. In addition, the patient was able to discontinue use of antidepressant medication after chelation.

**Discussion:**

A high index of suspicion of metal toxicity is necessary among persons with characteristic symptoms and signs in the absence of occupational exposure. Despite limited evidence for chelation in adults and in those with modest blood lead levels, this patient appeared to benefit from repeated chelation therapy. Both allopathic and alternative medicine practitioners and public health specialists need to be aware of the potential for contamination of and side effects from alternative pharmacologic and herbal therapies.

## Background

Ayurvedic medicines consist of herbs that are often intentionally combined with metals, such as lead, mercury, iron, and zinc, due to the belief that these metals contribute to their therapeutic effect [1]. Several studies have shown that approximately 20% of the Ayurvedic supplements (of both US and Indian origin) contain potentially toxic concentrations of several toxic metals [1,2]. These studies included supplements purchased via the internet [1] as well as supplements sold in stores in the U.S. [2]. Ayurvedic textbooks describe ancient protocols that Ayurvedic practitioners believe can purify the heavy metals, thereby eliminating their toxicity prior to consumption by humans [3]. Contrary to this belief, symptomatic lead toxicity following use of Ayurvedic medications has been reported repeatedly [4-6].

Results of a World Health Organization survey indicated that about 70-80% of the world populations rely on non-allopathic or traditional medicines, mainly of herbal sources, in their healthcare [5]. This widespread use of herbal medications, combined with the fact that many cases of lead toxicity may not be recognized clinically, makes contamination of such herbal medication with metals and other toxicants a serious global public health concern. Practitioners providing services to populations using non-conventional medicines should have a high index of suspicion of metal toxicity when confronted with one or more known adverse effects of metal ingestion. Otherwise, appropriate treatment may be delayed while unnecessary and invasive evaluations are performed.

Contributing to the failure to consider environmental sources of lead exposure is the fact that 95% of recognized adult lead toxicity is of occupational origin [7]. Other environmental sources of adult lead exposure include leaded gasoline (outside of the US), close proximity to lead emitting industries, home renovation, and numerous hobbies, including indoor shooting, use of lead containing solders, and lead containing ceramic glazes [8]. For the patient presented here, unnecessary tests and a lengthy delay in treatment could have been avoided if the initial treating clinicians had obtained a more complete environmental history including use of alternative medications and therapies. In addition, the identification of one case of heavy metal toxicity of a user of non-conventional medicines should be considered presumptive evidence of additional unidentified cases of toxicity and should trigger additional investigation.

## Case presentation

A 69 year old Caucasian male retired lawyer presented for evaluation of lethargy, fatigue, memory impairment, generalized weakness, severe constipation, anorexia, and weight loss of 18 kg over the preceding eight months. Approximately six weeks before the onset of his symptoms, he began using an Ayurvedic herbal medication, “Bhasma”, which he obtained while traveling in India. The medication was used to treat partial aphasia, cognitive impairment, and right-sided motor weakness resulting from a spontaneous left temporoparietal hemorrhagic stroke experienced two years previously.

Prior to presentation at our hospital, the patient had been admitted to another facility for evaluation of his symptoms and was found to have a hemoglobin level of 6.4 gm/dL (reference range: 13.2 gm/dL – 17.7 gm/dL). CT scan of the abdomen and pelvis, upper endoscopy and colonoscopy revealed no etiology for his anemia at that hospital. Subsequent to starting the Bhasma, the patient was also treated with citalopram (a selective serotonin reuptake inhibitor antidepressant) for depressed mood. He had no prior psychiatric history nor tendency toward depressed mood.

On further laboratory evaluation, a blood lead level (BLL) of 94 μg/dL (reference range: 0.0-4.9 μg/dL) was obtained. After the family suggested the Ayurvedic “Bhasma” medication could be the cause, it was analyzed for heavy metals and found to contain 19,400 mg/kg of lead and 1,430 mg/kg of arsenic. At the recommendation of the Iowa Poison Control Center, he was admitted to the medical intensive care unit for monitoring, given intramuscular chelation with Dimercaprol (BAL) at a dose of 5 mg/kg q4 hrs for three days, and given intravenous chelation with calcium disodium EDTA at a dose of 1500 mg/m2/day as a continuous infusion for five days beginning after the second dose of BAL. While in the ICU, he was monitored with daily labs including a CBC with differential, electrolyte panel including BUN and creatinine, calcium, phosphorous, magnesium, alkaline phosphatase, AST, ALT, glucose, and urinalysis. The patient’s blood lead level following two days of chelation with BAL and EDTA as described above had dropped to 27.1 μg/dL.

After six days, the patient was discharged from the hospital. His blood lead level on discharge was 25.5 μg/dL and he was instructed to complete a nineteen day course of oral chelation with dimercaptosuccinic acid (DMSA), 10 mg/kg three times daily for five days, then 10 mg/kg twice daily for fourteen days.

After completion of the nineteen day course of DMSA, the patient’s BLL had decreased to 23 μg/dL and he reported feeling much more energetic. Over the next six weeks his BLL rose to 38 μg/dL and the patient reported a coincident decline in speech fluency and memory. Additional chelation was recommended and he was treated with another course of oral DMSA which reduced his BLL to 20 μg/dL. The patient again reported that his mood was much improved and he then discontinued use of antidepressant medication without recurrence of depressed mood. Three months later, he received an alternative treatment at an Ayurvedic Health Clinic due to continued symptoms of cognitive impairment. The alternative treatment consisted of thirteen days of “Bastis” (enemas) and oil massages which were intended to rid his body of lead.

Six months after initial diagnosis and after having completed four courses of chelation therapy and an alternative treatment, the patient and his wife reported that he had persistent difficulty with “mental focus and clarity” in comparison to his post-stroke baseline. Repeat BLL was 29 μg/dL at that time. Although not typically offered to patients with a BLL below 40 μg/dL, a third nineteen day course of oral DMSA chelation therapy was administered in an effort to treat the patient’s cognitive symptoms. His blood lead level following the third course of chelation with DMSA was 7 μg/dL. Neuropsychological testing was performed both before and immediately following completion of the third course of DMSA to assist in distinguishing reversible neurocognitive effects of lead from fixed residual effects of his prior stroke. His clinical presentation was most notable for dysfluent aphasia referable to his cerebrovascular accident in 2009. Impairments were found in aspects of language, working memory/executive functions, and right-sided fine motor dexterity. He was oriented to time, personal information, and place. Working memory for digit sequences and geometric figures was low average to average. Visual processing speed was average for symbol discrimination and coding tasks. Speeded visuomotor sequencing was average under focused attention, but mildly impaired under divided attention. Verbal sequencing was borderline impaired. Letter-word verbal fluency was borderline impaired. Fine motor coordination was moderately impaired for dominant right hand and borderline impaired for non-dominant left hand.

The post chelation test showed “modest performance gains across various tests of attention and cognitive efficiency”. Specifically his performance on the Controlled Oral Word Association Test revealed a doubling of his raw score from 11 to 22 reflecting a significant improvement in semantic and phonetic verbal fluency. In addition his performance on the Wechsler Adult Intelligence Scale test revealed more than doubling of the Digit Span score from 3 to 7 and more modest improvements in the Symbol Search and Coding scores from 7 to 9 and 8 to 10, respectively. Although it is possible that practice effects contributed to the observed improvement, the patient reported a reduction in fatigue and greater clarity of his thinking after this third course of DMSA chelation suggesting at the least subjective clinical improvement.

## Discussion

This patient’s illness raises several issues relevant to environmental health specialists, clinical toxicologists and occupational health physicians who provide services to individuals and populations at risk of lead exposure from occupational or environmental exposures such as imported traditional or alternative herbal products or medicinal agents. These issues include contamination of alternative agents with metals and other toxicants, cognitive and affective benefits of treatment of lead toxicity among adult patients, and the risks and potential benefits of multiple chelation treatments.

### Clinical manifestations of adult lead toxicity

Adult lead toxicity can be categorized as either acute or chronic. Acute lead toxicity results from short-term, high dose lead absorption. Signs and symptoms often include normocytic or microcytic anemia, abdominal pain and constipation (“lead colic”), arthralgias and myalgias, and central nervous system impairment including headache, mood disorder and encephalopathy and can occur within weeks of the onset of sufficient exposure [7,9]. Chronic toxicity from lower doses of lead absorbed over longer durations results in more gradual increase in body burden and more variable symptoms, including decreased libido, impotence, infertility, anorexia, abdominal pain, weight loss, change in bowel habits, muscle weakness and pain, fatigue, depression, irritability, insomnia, paresthesias, headache, and nervousness [10], some of which were reported by our patient. Dose-related effects of lead on multiple organ systems have been described, including hematologic abnormalities such as normocytic or microcytic anemia, neurologic effects including peripheral nerve dysfunction and encephalopathy, renal toxicity including interstitial nephritis and elevated blood pressure, and reproductive dysfunction such as infertility and elevated miscarriage rates [8].

Our patient manifested many of the known symptoms and signs of acute lead toxicity, including anemia, abdominal pain and constipation, altered affect, and neuro-cognitive impairment [11]. Given onset of symptoms six weeks after initiating Ayurvedic treatment, he likely experienced relatively rapid increase in blood lead levels. As expected, his blood lead levels decreased immediately following chelation therapy and showed some rebound after completion of treatment suggesting re-distribution from bone.

### Risks and benefits of chelation treatment

In the current era, the most commonly recommended agents for chelation of lead are calcium-disodium EDTA and DMSA. DMSA has the advantage of enteral administration whereas EDTA is routinely administered intravenously. While chelation with either agent is considered relatively safe [12], known side effects of each should be considered prior to administering treatment. Adverse effects of DMSA therapy include elevation of hepatic transaminases and mucocutaneous and skin reactions [13]. While moderate elevation of hepatic transaminases are not considered a contraindication to initiation of chelation with DMSA [13], the risks and benefits of chelation in the presence of liver disease or prior DMSA-related skin reactions should be considered when choosing a chelating agent. Persons with glucose-6-phosphate dehydrogenase deficiency should not receive DMSA as the drug has been reported to cause hemolytic anemia in these patients [14]. Further, DMSA has been shown to produce both decreased maternal weight gain and fetotoxicity in experimental studies [13]. Although these effects were produced at higher doses than are used clinically, IV EDTA is generally considered a safer alternative if chelation is necessary during pregnancy.

The most serious side effect of EDTA chelation is dose-dependent nephrotoxicity [15] and close monitoring of renal function should be performed routinely in patients undergoing EDTA therapy. While skin reactions have also been described with use of EDTA, it is hypothesized that these mild reactions are due to zinc deficiency resulting from chelation rather than an allergic reaction [15].

Chelation decisions are complicated by the fact that symptoms of lead toxicity are variable and do not consistently associate with observed blood lead levels. Adults with characteristic symptoms and substantially elevated blood lead levels typically report symptomatic improvement following chelation therapy. Furthermore, chelation is especially recommended among persons with elevated blood lead levels and altered mental status suggestive of encephalopathy [12]. However, evidence for chelation is sparse for adult patients with blood lead levels below 30 μg/dL, regardless of symptoms, and for asymptomatic patients with blood lead levels above 30 μg/dL. In this setting of uncertainty, we elected to chelate the case patient when his BLL was 29 μg/dL in an effort to more fully control his neurocognitive and affective symptoms. One consideration was the potential that his symptoms and cognitive impairment were residual from his prior CVA and would not be responsive to additional chelation. However, this patient provided a clear history of greater cognitive symptoms following use of the Ayurvedic medication in comparison to his post stroke (but pre-medication) baseline, suggestive of a toxic etiology.

We based the decision to chelate on epidemiological evidence associating relatively low BLLs to adverse cognitive effects and a small number of case reports of improvements in cognitive and affective symptoms following chelation therapy among patients with low blood lead levels. Adverse neurocognitive effects among adults with BLLs below 40 μg/dL have been described [16-19]. Epidemiologic evidence of decrements in cognitive function among adults has been reported for mean blood lead levels of 4.5 μg/dL [20]. Thus, it is plausible that the case patient’s symptoms and cognitive impairments were, at least in part, related to lead exposure.

In addition to epidemiological evidence of neurological effects at low BLLs, apparent reversibility of cognitive impairment at low BLLs has been reported in several case reports. Linz et al. [21] described the effects of chelation treatment of a 37 year old construction worker with a BLL of 16 μg/dL. One year prior, he had a BLL of 109 μg/dL which decreased spontaneously after removal from exposure. Despite his BLL reduction, he reported persistent symptoms of short term memory loss, insomnia, fatigue, weakness and depression. Neuropsychological testing was administered before and following chelation with EDTA. Mild to moderate improvement was noted on tests of verbal fluency, visual motor speed, and overall memory. In addition, a standard test of depression showed improvement. Frumkin and Gerr [22] reported a substantial improvement in mood in a lead battery plant worker following chelation with DMSA. The patient had been given a diagnosis of major depression and was treated with fluoxetine. The patient’s BLL was 26 μg/dL immediately prior to chelation and was 12 μg/dL following chelation. The patient was able to discontinue fluoxetine after treatment and reported improvement in mood, increased restfulness of sleep, and reduction in irritability.

Clearly, the evidence base is limited regarding treatment of patients with modest elevations in BLL and persistent neurocognitive complaints. Treatment decisions should be made using the best available evidence, individualized to the patient’s symptoms and situation, and in consultation with the patient. In the case presented here, the patient was frustrated by his ongoing cognitive difficulties and also reported a strong desire to discontinue use of antidepressant medication. The relatively small risk of adverse events from a third course of DMSA chelation was outweighed by the possibility of improved mood and cognitive performance. His cognitive function improved following treatment and his symptoms of depression resolved completely, as described previously [22,23].

## Conclusions

The workplace is the most common source of adult lead exposure. However, non-occupational sources of lead must be considered among patients with characteristic signs and symptoms of lead toxicity and no known occupational exposure. It is axiomatic that effective management of lead toxicity requires identification and mitigation of the exposure.

The case presented here raises concern that the ready availability of unregulated domestic and foreign herbal medications and supplements is a potential public health hazard. A 2007 National Health Interview Survey found that 38% of adults in the U.S. use complementary and alternative therapies [24]. Despite this widespread use, the alternative medicine industry is, essentially, self-regulating and without federal enforcement. Recommendations have been made by the U.S. Food and Drug Administration that manufacturers of supplements sold in the U.S. conform to “Good Manufacturing Practices” [25], but this case report as well as others demonstrates that more regulation is necessary to ensure the safety of persons consuming these therapies. The sentinel case presented here has led to an ongoing investigation by state public health authorities of other users of Ayurvedic supplements.

Another question raised by this case is that of when and whether chelation is likely to benefit adult patients with modest elevations of BLL. We know of no consensus guidelines for administration of chelation therapy to such adults. The case presented here illustrates the potential benefit of chelation on cognitive function among chronically exposed adults, but provides no definitive guidance. A sufficiently large intervention study of the benefits of chelation on cognitive impairment (and other adverse effects of lead) among patients with modestly elevated BLLs would be useful to clinicians. A risk-benefit analysis incorporating the known adverse effects of chelation therapy, improvements in subclinical cognitive function attributable to lowering body lead burdens, and the cost of chelation therapy would support evidence-based guidelines for management of mild to moderate elevations of BLL among adults.

An important lesson learned from this case is that prevention, recognition, and treatment of lead toxicity requires a high index of suspicion. Both allopathic and alternative medicine practitioners and public health specialists need to be aware of the widespread use of herbal and alternative therapies and the potential for contamination of and side effects from these therapies. Additionally, although not evident in this case, concomitant use of alternative remedies with traditional pharmacologic medications could produce previously unknown adverse interactions. Recognizing that no therapeutic agent ingested by humans can be considered completely “safe”, and that both risks and benefits should be considered, is an important lesson for both practitioners and patients, alike.

## Consent

Written informed consent was obtained from the patient for publication of this case report. A copy of the written consent is available for review by the Editor-in-Chief of this journal.

## Competing interests

The authors declare that they have no competing interests.

## Authors’ contributions

LF facilitated blood lead testing and chelation for the patient. LF and LB participated in follow-up care for the patient. All authors collaborated in drafting the manuscript. All authors have read and approved the final manuscript.
